# SARS-CoV-2-Associated Obliterative Arteritis Causing Massive Testicular Infarction

**DOI:** 10.3390/clinpract11020037

**Published:** 2021-05-06

**Authors:** Dámaso Parrón, Ane Gartzia, Ane M. Iturregui, Igone Imaz, Claudia Manini, Jorge García-Olaverri, José I. López

**Affiliations:** 1 Department of Pathology, Cruces University Hospital, Barakaldo, 48903 Bizkaia, Spain; damaso.parroncollar@osakidetza.eus (D.P.); ane.gartziarivero@osakidetza.eus (A.G.); mirenigone.imazmurga@osakidetza.eus (I.I.); 2 Department of Urology, Cruces University Hospital, Barakaldo, 48903 Bizkaia, Spain; anemiren.iturreguidelpozo@osakidetza.eus (A.M.I.); jorge.garcia-olaverrirodriguez@osakidetza.eus (J.G.-O.); 3 Department of Pathology, San Giovanni Bosco Hospital, 10154 Turin, Italy; claudiamaninicm@gmail.com; 4 Biocruces-Bizkaia Health Research Institute, Barakaldo, 48903 Bizkaia, Spain

**Keywords:** SARS-CoV-2, COVID-19, arteritis, pathology, testicular infarction

## Abstract

A 26-year-old man with symptomatic SARS-CoV-2 infection developed a sudden-onset acute testicular pain. The echo-doppler images showed massive testicular infarction, so orchiectomy was performed. On gross examination, the surgical specimen showed complete testicular necrosis and diffuse thickening of the testicular coverings. Under the microscope, a severe obliterative arteritis was evidenced. SARS-CoV-2 spike antibody was detected by immunohistochemistry in the arterial endothelium. Electron microscopy displayed intracytoplasmic spiky viral particles in endothelial cells. The patient was treated with corticoids and was asymptomatic at last contact.

## 1. Introduction

Individual host reactivity against SARS-CoV-2 is largely unpredictable and responsible for most symptoms and sequelae of this infection. Vasculitis is a systemic response to the virus with variable qualitative and quantitative clinical manifestations. Here, we present an extreme case of massive unilateral testicular necrosis occurring in the recovery phase of SARS-CoV-2 infection in a young man, a manifestation which represents a previously non reported clinical complication of this infection.

## 2. Case Report

**Figure 1:**
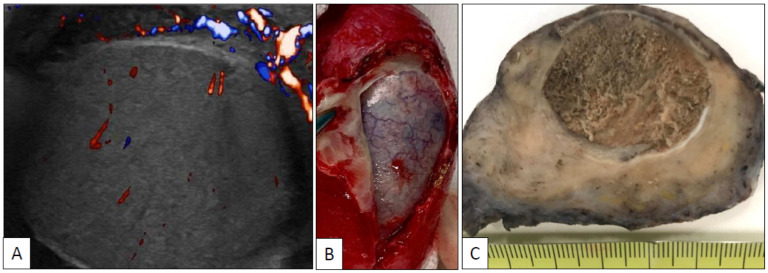
Echo-doppler of the left testicle shows diffuse ischemic infarction (**A**); left orchiectomy specimen (**B**) showing complete testicular necrosis and thickening of the spermatic cord and peri-testicular coverings (**C**).

A 26-year-old man with symptomatic SARS-CoV-2 infection, including mild dyspnea and fever, developed a left testicular pain two days after the acute symptomatic infection was vanished (PCR negative, IgG positive). No antecedents of any systemic disease were recorded in the anamnesis and analytical data, other than those specifically related to the SARS-CoV-2, were within the normal limits. No signs of coagulopathy were detected. The echo-doppler study of the testis at that time showed mild hypoperfusion. Testicular pain was then thought to be secondary to orchitis and symptomatic therapy with empiric antibiotic coverage was started. Pain, however, did not recover and 3 days after a second echo-doppler showed a complete testicular infarction (Figure 1A). Left orchiectomy was then performed (Figure 1B). No signs or symptoms of vasculopathy were detected elsewhere. The patient was treated with corticoids and was asymptomatic at last contact. Right testis is not affected.

**Figure 2:**
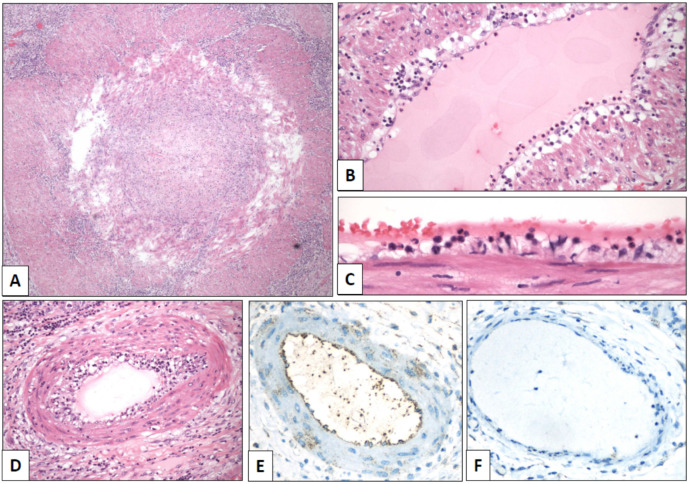
Spermatic cord arteries show temporal heterogeneity of the lesions, from severe luminal occlusion ((**A**), original magnification, ×40) to mild endotheliitis with luminal polymorphonuclear elements ((**B**), ×100) and endothelial tumefaction ((**C**), ×640). Moderate lesions showed endothelial thickening with mixed inflammatory infiltrates ((**D**), ×240). Positive immunostaining with SARS-CoV-2 spike antibody was detected in endothelial cells of arteries (((**E**), ×400) with a negative control in a vein of the same paraffin block ((**F**), ×400)).

Grossly, the testicle (length of the surgical specimen: 8 cm, testicular size: 3 cm, surgical specimen weight: 73 g) showed a massive infarction and diffuse thickening of the spermatic cord and testicular coverings (Figure 1C). A segmentary obliterative endotheliitis affecting large, medium, and small-sized arteries was observed in the spermatic cord. Vascular injury was heterogeneous in intensity and distribution. The more severely damaged vessels, including the spermatic artery, displayed occlusive endothelial proliferation (Figure 2A) admixed with neutrophils, eosinophils, and lymphocytes. Less damaged vessels showed endothelial tumefaction and vacuolization with associated inflammation (Figure 2B–D). Additionally, there were well-preserved arteries and veins without any apparent morphological alteration. Vascular lumina showed only very occasional thrombotic phenomena.

**Figure 3:**
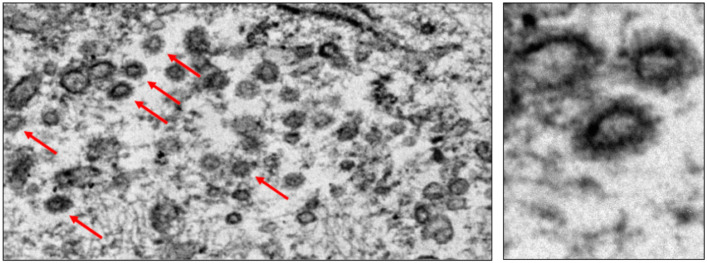
Spiked viral particles were identified within the cytoplasm of endothelial cells.

The intravascular lymphocyte population associated with endothelial damage included both CD4 and CD8 cells. Interstitial and perivascular infiltrates were composed of a mixture of macrophages, and T and B cells. Immunohistochemistry against the SARS-CoV-2 spike protein (Gene Tex, clone 1A9, dilution 1:500) showed positive endothelial reaction (Figure 2E, negative control in Figure 2F). Moreover, viral spiked particles were detected in endothelial cells (Figure 3). Testicular parenchyma was diffusely necrotic.

## 3. Discussion

COVID-19-associated vasculopathy has been well documented in almost any organ [1] being responsible for most of the symptoms and sequelae of this infection. Direct endothelial damage induced by the virus alters the in situ glycocalyx thus favoring hypercoagulability [2]. Diffuse alveolar damage, interstitial, and organizing pneumonia have been observed in the lungs of several autopsy studies [3]. However, almost every organ can be affected. Some other similar examples of ischemic changes secondary to virus-related vasculopathy, i.e., ischemic vascular stroke [4], intestinal ischemia [5], and gallbladder ischemic necrosis [6], some of them occurring in young people, have been occasionally reported. Orchitis and impaired spermatogenesis have been previously associated with COVID-19 infection [7,8]. However, massive testicular ischemic infarction in the context of COVID-19 infection has not been reported so far.

The present case recalls the attention of general practitioners and urologists when facing testicular pain in the context of SARS-CoV-2 infection.
